# Telomerase peptide vaccination of patients with non-resectable pancreatic cancer: a dose escalating phase I/II study

**DOI:** 10.1038/sj.bjc.6603437

**Published:** 2006-10-24

**Authors:** S L Bernhardt, M K Gjertsen, S Trachsel, M Møller, J A Eriksen, M Meo, T Buanes, G Gaudernack

**Affiliations:** 1 Ullevål University Hospital, Oslo, Norway; 2 Section for Immunotherapy, Institute for Cancer Research, The Norwegian Radium Hospital, Oslo, Norway; 3 GemVax AS, Oslo, Norway

**Keywords:** telomerase vaccine, immunotherapy, inoperable pancreatic cancer

## Abstract

Patients with inoperable pancreatic cancer have a dismal prognosis with a mean life expectancy of 3–6 months. New treatment modalities are thus urgently needed. Telomerase is expressed in 85–90% of pancreas cancer, and immunogenic telomerase peptides have been characterised. A phase I/II study was conducted to investigate the safety, tolerability, and immunogenecity of telomerase peptide vaccination. Survival of the patients was also recorded. Forty-eight patients with non-resectable pancreatic cancer received intradermal injections of the telomerase peptide GV1001 at three dose levels, in combination with granulocyte–macrophage colony-stimulating factor. The treatment period was 10 weeks. Monthly booster vaccinations were offered as follow-up treatment. Immune responses were measured as delayed-type hypersensitivity skin reaction and *in vitro* T-cell proliferation. GV1001 was well tolerated. Immune responses were observed in 24 of 38 evaluable patients, with the highest ratio (75%) in the intermediate dose group. Twenty-seven evaluable patients completed the study. Median survival for the intermediate dose-group was 8.6 months, significantly longer for the low- (*P*=0.006) and high-dose groups (*P*=0.05). One-year survival for the evaluable patients in the intermediate dose group was 25%. The results demonstrate that GV1001 is immunogenic and safe to use. The survival data indicate that induction of an immune response is correlated with prolonged survival, and the vaccine may offer a new treatment option for pancreatic cancer patients, encouraging further clinical studies.

Pancreatic cancer is a highly lethal disease and little therapeutic progress has been achieved in the last decades. Vaccination against cancer is currently tested in many clinical trials as a new treatment modality. In pancreatic cancer, few molecularly characterised antigens have so far been available for use in vaccines. However, the catalytic subunit of telomerase, hTERT, expressed in 85–90% of human cancer tissues (Vasef *et al*, 1999) and an attractive ‘universal’ tumour antigen (Autexier, 1999), is also expressed in pancreatic cancer where it has been used diagnostically (Suehara *et al*, 1998; Uehara *et al*, 1999). By turning on hTERT and telomerase activity, cancer cells are enabled to maintain functional telomeres at the end of chromosomes, and are prevented from going into senescence. Telomerase is consequently a key enzyme in the process of immortalisation of cancer cells and has a pivotal role in carcinogenesis.

It is well established that T cells of the human immune system can recognise hTERT peptides, and a number of HLA-class I and class II epitopes have been characterised (Vonderheide *et al*, 1999; Minev *et al*, 2000; Arai *et al*, 2001; Vonderheide *et al*, 2001; Hernandez *et al*, 2002; Scardino *et al*, 2002; Schroers *et al*, 2002, 2003; Gross *et al*, 2004). Recently it has also been reported that telomerase-specific CD4+ and CD8+ T-cell responses are induced upon vaccination with hTERT-transfected dendritic cells (Su *et al*, 2005). Vaccination with the telomerase peptide GV1001 was recently shown to induce T-cell responses in patients with non-small cell lung cancer (Brunsvig *et al*, 2006).

hTERT is expressed in some normal tissues, including embryonic cells, bone marrow stem cells (Uchida *et al*, 1999) and epithelial cells in colonic crypts (Tahara *et al*, 1999). Safety is therefore an important issue in the development of telomerase-targeted therapies. In general, telomerase activity is highly overexpressed in malignant cells, compared to normal cells (Tahara *et al*, 1999). This may provide the necessary window of opportunity for the immune system to discriminate between tumour cells and normal cells. Previous reports indicate that telomerase-specific T cells capable of killing telomerase-positive target cells did not kill CD34+ bone marrow stem cells (Vonderheide *et al*, 1999), and that induction of telomerase-specific T-cell responses in cancer patients is safe (Parkhurst *et al*, 2004; Vonderheide *et al*, 2004; Su *et al*, 2005).

Based on our earlier experience with vaccination with mutant RAS peptides (Gjertsen *et al*, 2001), a protocol for telomerase peptide vaccination of patients with non-resectable pancreatic cancer was developed. The telomerase peptide used in this study, GV1001; hTERT (611–626) is a promiscuous HLA class II epitope, a property making GV1001 a candidate for a general cancer vaccine that may, in contrast to many other peptide-based vaccines, be administered without prior HLA typing of the patients. Most tumours do not express HLA class II molecules on their surface but HLA class II restricted CD4+ T cells are nevertheless considered to be mandatory for establishing and maintaining a clinically efficient anticancer immune response (Mumberg *et al*, 1999; Schurmans *et al*, 2001).

The objectives of the present clinical trial and for the follow-up protocol were to investigate the safety and tolerability of GV1001 administration, to determine the optimal dose for immunological response, and register survival.

## PATIENTS AND METHODS

### Patient selection

Forty-eight treatment naive patients with non-resectable, histologially confirmed adenocarcinoma of the pancreas were enrolled in the study (September 2000–March 2003). Karnofsky performance status was >70%. Bone marrow, liver, heart and renal function adequate, and age range 18–75 years. Forty-eight patients were enrolled and sequentially divided into three groups, given either a low dose (*n*=11), an intermediate dose (*n*=17), or a high dose of the vaccine (*n*=20). For details see Table 1.

Patients who received at least six vaccinations over 4 weeks were considered evaluable for assessment of immune responses and survival. The distribution of the evaluable patients was eight, 16, and 14 in the low-, intermediate-, and high-dose groups, respectively. The trial was approved by the regional ethics committee and performed in compliance with the Declaration of Helsinki Principles.

### Vaccine and control peptides

**Figure 1:**
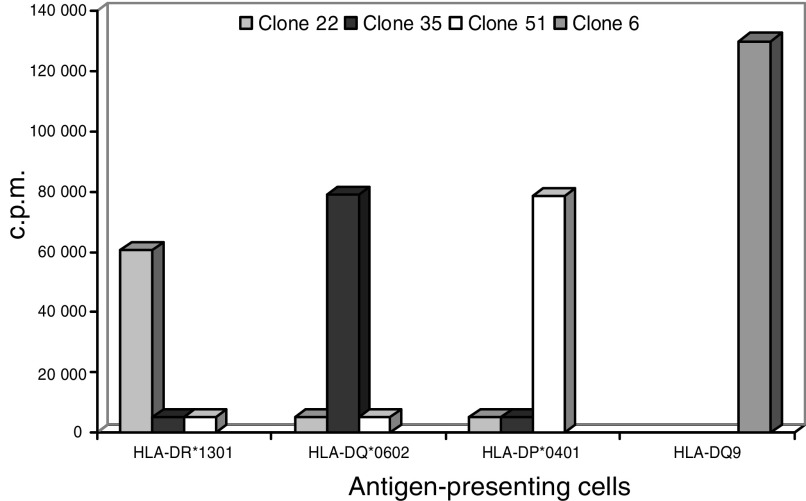
*In vitro* stimulation of GV1001-specific T-cell clones from patient no 102 (clone 22) and 105 (clones 6, 35, 51). Clones were stimulated with GV1001 (25 *μ*M) pulsed onto APCs homozygous for different HLA-class II molecules as indicated. Results are presented as c.p.m.

The vaccine is a sterile lyophilised product consisting only of the synthetic peptide GV1001; EARPALLTSRLRFIPK, corresponding to the hTERT (611–626) fragment. GV1001 is a promiscuous HLA class II epitope (Figure 1) and was selected after a preclinical screening program. GV1001 was supplied by Avecia Biotechnology, Cheshire, England. The vaccine was manufactured in compliance with cGMP by Isopharma AS, Kjeller, Norway, and released for clinical use by GemVax AS, Porsgrunn, Norway. Two synthetic peptides, PEP 544; LMSVYVVELLRSFFYVTE (hTERT (548–566)) and PEP 508; LLDILDTAGHEEYSAMRDQ (KRAS (52–70) Q61H), were used as negative controls in the *in vitro* T-cell proliferation assays used to monitor T-cell response in blood samples from the vaccinated patients. The control peptides were supplied by The Corporate Research Centre, Norsk Hydro, Porsgrunn, Norway.

### Recombinant human telomerase reverse transcriptase

Recombinant hTERT (563–735) was cloned in frame with the N-terminal 6 × His tag in *Escherichia coli* expression vector pET28b(+) (Novagen, Darmstadt, Germany). The protein was produced in *E.coli* BL21 Codon Plus (DE3)-RIPL (Stratagene, La Jolla, CA, USA), purified by NiNTA chromatography under denaturing conditions (Qiagen, Hilden, Germany) and tested by Western blot analysis with anti-His antibodies (Qiagen) and Rabbit anti-Mouse Ig HRP (DakoCytomation, Glostrup, Denmark). The positive fraction was dialysed in Slide-A-Lyzer® Dialysis Casette 10000 MWCO (Pierce, Rockford, IL, USA) against MQ-water and sterile filtered before use.

### Treatment protocol

A comprehensive assessment of adverse drug reactions, blood screening, physical examination, and assessment of Karnofsky performance status was performed at baseline and at each visit.

The vaccine was administered by intradermal (i.d.) injection in the right para-umbilical area following the schedule; three injections in week 1 (Monday, Tuesday, and Friday) and one weekly injection in weeks 2, 3, 4, 6, and 10. The three different doses of vaccine administered were; *low dose*: 60 nmole (112 *μ*g) GV1001 in 0.10 ml saline, *intermediate dose*: 300 nmole (560 *μ*g) GV1001 in 0.125 ml saline, and *high dose*: 1.0 *μ*mole (1.87 mg) GV1001 in 0.20 ml saline. From 5 to 15 min before each vaccine injection, 30 *μ*g granulocyte–macrophage colony-stimulating factor (Leucomax®; Schering-Plough, Cork, Ireland) in 0.10 ml saline was injected i.d. at the vaccination site. After finishing the primary protocol eighteen evaluable patients entered the follow-up study. Monthly booster vaccination was offered, for up to 1 year starting with patient no. 12 in the low-dose group. The patients were given vaccine doses identical to the ones they received in the initial protocol. The follow-up treatment was given to patients in good general health.

### Delayed-type hypersensitivity

Delayed-type hypersensitivity (DTH) skin test was performed at visit one (baseline) and at all visits from week 2 and onwards. As DTH test 60 nmole (112 *μ*g) GV1001 in 0.10 ml saline was injected i.d. in the left para-umbilical area. A positive DTH test was defined as a ⩾5 mm diameter erythema/induration 48 h after the administration. The patients were instructed to measure and record the erythema/induration and report the result to the clinician.

### Monitoring of T-cell responses

At baseline and at weeks 6 and 10, 50 ml of Acid Citrate Dextrose-blood was sampled to assess changes in *in vitro* proliferative GV1001-specific T-cell responses. Proliferation was measured as count per minutes (c.p.m.) after incubation and uptake of ^3^H-thymidine in the standard proliferation assay described below. A stimulatory index (SI) (c.p.m. with GV1001 divided by c.p.m. without GV1001) ⩾2 was considered as a positive T-cell response to GV1001. PBMCs were isolated from peripheral blood using density centrifugation over Ficoll–Hypaque (Lymphoprep; Nycomed, Oslo, Norway), washed and frozen in RPMI-1640 (Gibco, Paisley, UK) with 20% FCS (PAA Laboratories GmbH, Paching, Austria) and 10% DMSO and stored in liquid N_2_. Following the inclusion of patient number 211, FCS was substituted by 100 mg ml^−1^ (10%) Human Albumin, owing to the generation of high background proliferative responses against antigens present in FCS. In the *in vitro* T-cell assay used here, specific T cells were expanded from PBMC by two cycles of antigen-driven stimulation before assaying. Samples harvested before and after vaccination were processed in parallel. No responses were observed in PBMC from normal blood donors using this procedure. Thawed PBMC were seeded at 1 × 10^6^ per well in 24-well plates (Costar, Cambridge, MA, USA) in 1 ml of RPMI-1640 containing 15% heat-inactivated human serum and antibiotics supplemented with GV1001 or control peptides at 25 *μ*M concentration. After 3 days of culture the medium was supplemented with 10 U ml^−1^ of recombinant human interleukin-2 (Amersham, Aylesbury, UK). Cultured cells were re-stimulated for one more cycle after 7–9 days of culture and finally tested on days 14–18 for specific proliferating capacity against GV1001 and control peptides at 25 *μ*M concentration, by using 5 × 10^4^ T cells and autologous, irradiated (30 Gy) PBMCs (5 × 10^4^ cells well^−1^) as antigen-presenting cells (APCs). After 2 days, wells were pulsed with 3.7 × 10^4^ Bq of ^3^H-thymidine over night and counted. Values are given as mean c.p.m. from triplicate wells. Cytokine production by responding T cells was measured in supernatants taken from T-cell assays 24 h after peptide stimulation, using a human 17-plex cytokine kit and the Bio-Plex instrument (BioRad Laboratories Inc., Hercules, CA, USA), as described by the manufacturer. Data are given as picogram cytokine ml^−1^ and are calculated based on standard curves for each of the cytokines. At baseline, responses against either purified protein derivative of *M. tuberculosis*; The Veterinary Institute, Oslo, Norway) or superantigen (SEC-3; Toxin Technology, Sarasota, FL, USA) were also assessed in a 7-day assay, without Il-2, as a measure of general immune status. GV1001-specific T cells from responding patients were cloned by limiting dilution as described previously (Gjertsen *et al*, 1997).

### Safety and toxicity

The patients were followed closely for signs of adverse events during and after each vaccination. Adverse events were recorded using the WHO toxicity criteria.

### Statistics/evaluable patients

Data from all patients who received GV1001 vaccination were included in the safety analysis. All patients that completed visit six (week 4) and complied with the inclusion criteria, were considered as evaluable and were included in the analysis of immune response and survival. Survival values are given as median and 95% confidence interval. Differences between survival of immune responders and non-responders, and between different treatment groups, were tested by log-rank test.

## RESULTS

A summary of individual patient's characteristics and results are given in Table 1.

### Adverse events

GV1001 was administered on an outpatient basis and was well tolerated in all the 48 patients that received at least one dose of vaccine. In total 424 vaccine doses were administered during this trial (1–21 doses per patient). In addition 354 DTH injections were given. No sign of toxicity or clinically severe adverse events related to the treatment was observed in any of the treated patients, including long-term survivors who received 14–21 injections. All patients reported at least one treatment related injection site disorder such as erythema or induration. Observed drug related side effects were fever (2%), chills (10%), pain (6%), fatigue (2%) nausea (12%), and vomiting (2%). The frequency of patients with adverse events in the three dose groups was five out of 11 (45%), seven out of 17 (41%), and two out of 20 (10%). The number of drug related adverse events were six, 22, and four in the low-, intermediate-, and high-dose group, respectively. The intermediate group received more injections and were followed up for a much longer period owing to their prolonged survival. This probably explains a higher total number of adverse events in this group.

### Characterisation of immune responses against GV1001

**Figure 2:**
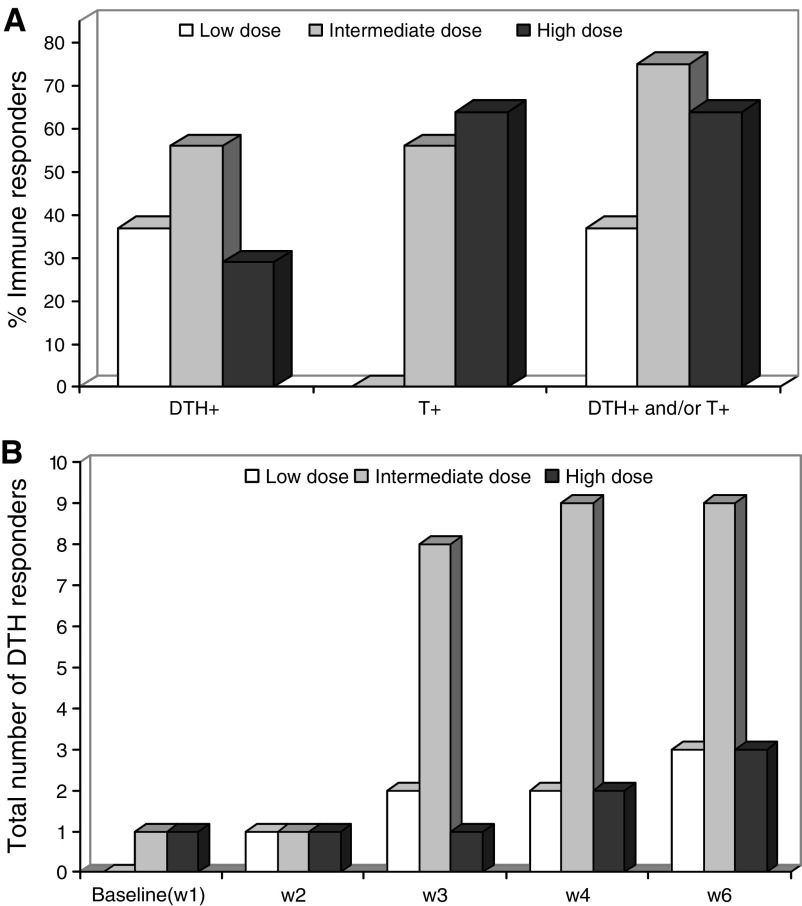
Type and percentage of immune responders in patients vaccinated with low, intermediate, and high dose GV1001 (**A**) and kinetics of immune responses in the three dose groups, as measured by DTH response (**B**). Bars show accumulated responses in each group.

**Figure 3:**
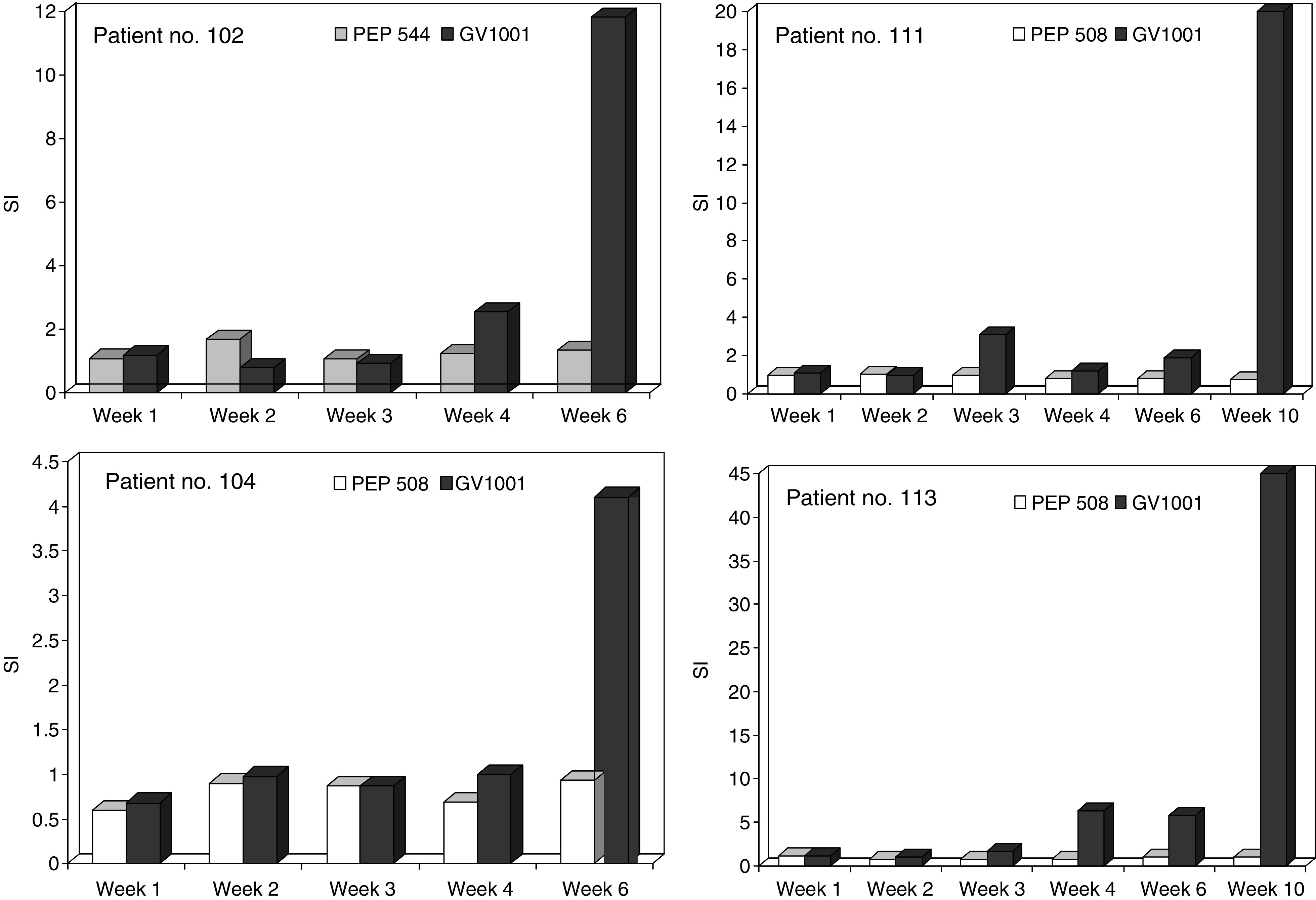
*In vitro* T-cell responses against GV1001 in blood samples harvested before (week 1), during and after vaccination (weeks 2, 3, 4, 6, and 10). Responses are given as SI. Control cultures were primed and stimulated with control peptide PEP 544 or PEP 508.

Patients with a positive DTH test or the presence of GV1001-specific T cells in peripheral blood after vaccination were considered as immune responders. Figure 2 shows that induction of detectable immune responses was dependent on vaccine dose. The highest percentage of responding patients was observed in the intermediate dose group, where 75% of the evaluable patients responded (Figure 2A). The difference in the number of immune responders observed in the three dose groups was not statistically significant. The results also indicate that detectable immune responses, as measured by DTH were induced more rapidly in the patients receiving the intermediate dose (Figure 2B). No *in vitro* T-cell responses were seen in the low-dose group (Table 1). The *in vitro* immune responses detected in the intermediate dose group were both more frequent and more vigorous than in those of the high-dose group (data not shown). Examples of the kinetics of the T-cell response, as measured by *in vitro* T-cell proliferation for four individual patients in the intermediate group are given in Figure 3. Enumeration of specific T cells is not possible with the approach used here and is complicated by the fact that a large number of different HLA class II molecules probably are involved. However, the requirement for *in vitro* antigen driven expansion before testing indicate that a relatively low number of GV1001-specific T cells were present in circulation at the time of harvesting of PBMC.

Overall, a vaccine related immune response was detected in 63% of the evaluable study population. Patient nos. 101 and 221 demonstrated a pretreatment immune response to GV1001, patient no. 101 with both DTH and T-cell response and patient no. 221 with DTH response.

**Figure 4:**
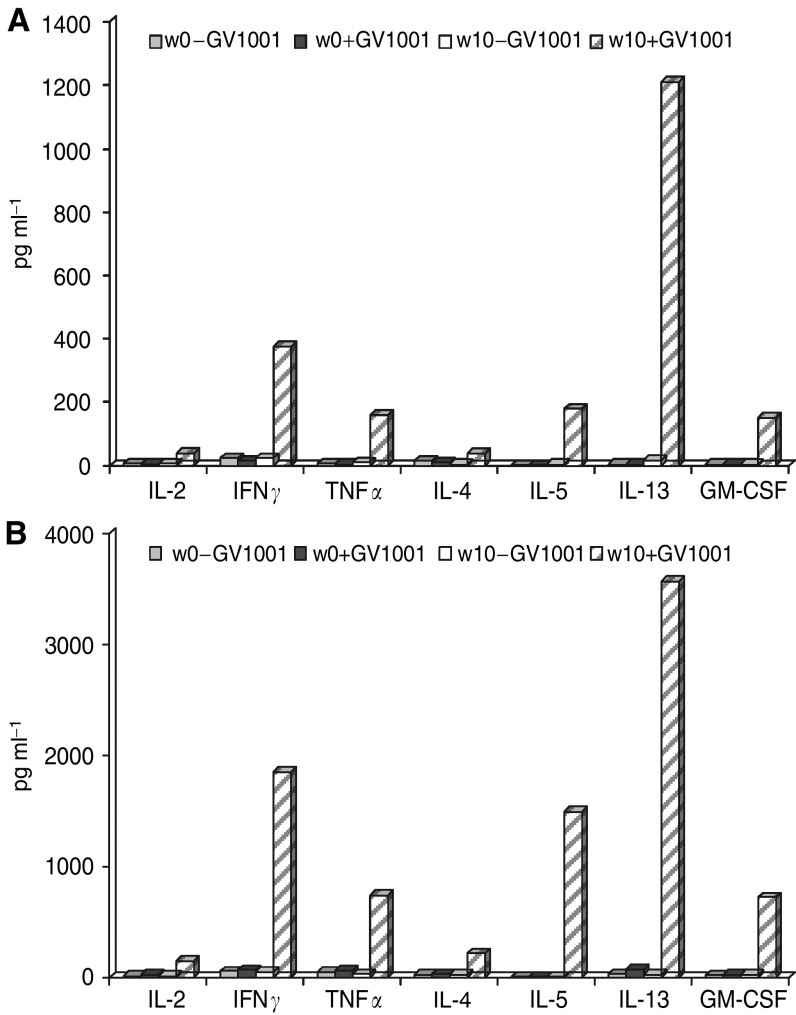
(**A**) Cytokine profile generated by stimulating PBMC from patient 101 in the intermediate dose group harvested before vaccination (W0) and on week 10 (W10) and (**B**) patient 212 in the high-dose group. Data are given as picogram cytokine ml^−1^ in supernatants harvested 24 h after stimulation of primed cells with APC with or without GV1001.

**Figure 5:**
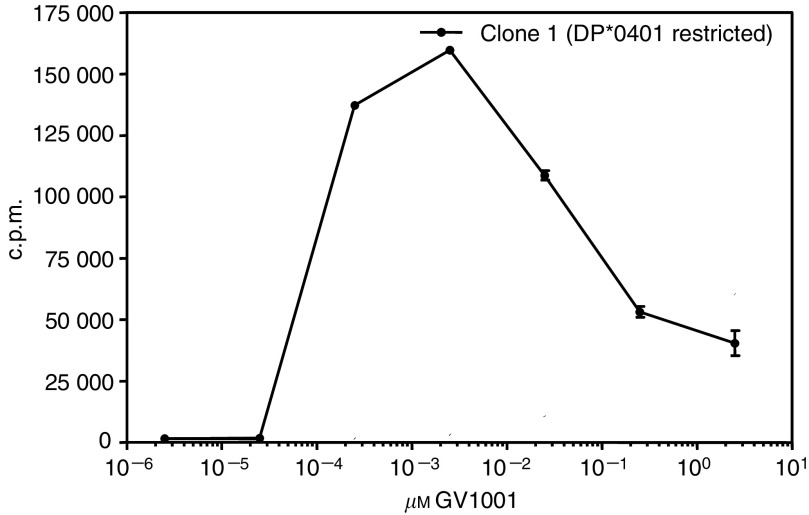
Dose response curve for T-cell clone 1 from patient no 102 stimulated with GV1001. Fifty thousand T cells were stimulated using 25 × 10^3^ autologous EBV-transformed B cells as APCs. Results, counted after 3 days, are presented as mean c.p.m. of triplicates±s.d.. Similar bell shaped curves were observed with other T-cell clones.

The observed discrepancy between DTH reactions and T-cell responses *in vitro* might either reflect different sensitivities in the two assays or be a result of biologically different immune reactions being generated. Thus, a shift in cytokine production away from classical inflammatory cytokines might result in a lack of DTH reaction. To further address the latter possibility, we first investigated the cytokine profile generated against GV1001 by peripheral blood T cells from two patients with different immune reactions. Patient 101 from the low-dose group was DTH-positive, but no proliferative response was detected in peripheral blood. Patient 212 from the high-dose group was DTH-negative but mounted a T-cell response *in vitro*. Data recorded in Figure 4A and B demonstrate that cytokines are specifically produced upon stimulation with GV1001 in samples harvested in week 10 following vaccination in both patients. Only background levels were seen in prevaccine samples. Although the cytokine levels in the sample without detectable proliferation was lower, the cytokine profiles of the two samples were identical. Interestingly, both patients displayed a broad profile with cytokines belonging to both the Th1 and Th2 type. This indicates that the absence of a DTH reaction in patient 212 is not owing to a switch from a Th1 profile to a Th2 profile. To further investigate the relationship between GV1001 dose and T-cell response, T cells from one responding patient were cloned (patient no. 102). As demonstrated in Figure 5, the proliferative response of the CD4+ Th clone TLC1 is represented by a bell-shaped curve peaking at a GV1001 concentration of 1.5 nM of GV1001. At 1000 nM, the response was reduced to about 20% of the peak response, indicating that high GV1001 doses may result in tolerance or anergy induction. Identical results were obtained with several T-cell clones derived from patients with non-small cell lung cancer vaccinated with GV1001 (data not shown). Interestingly these T-cell clones use different T-cell receptors and are restricted by different HLA class II molecules, indicating that this is a general, rather than anecdotal finding.

**Figure 6:**
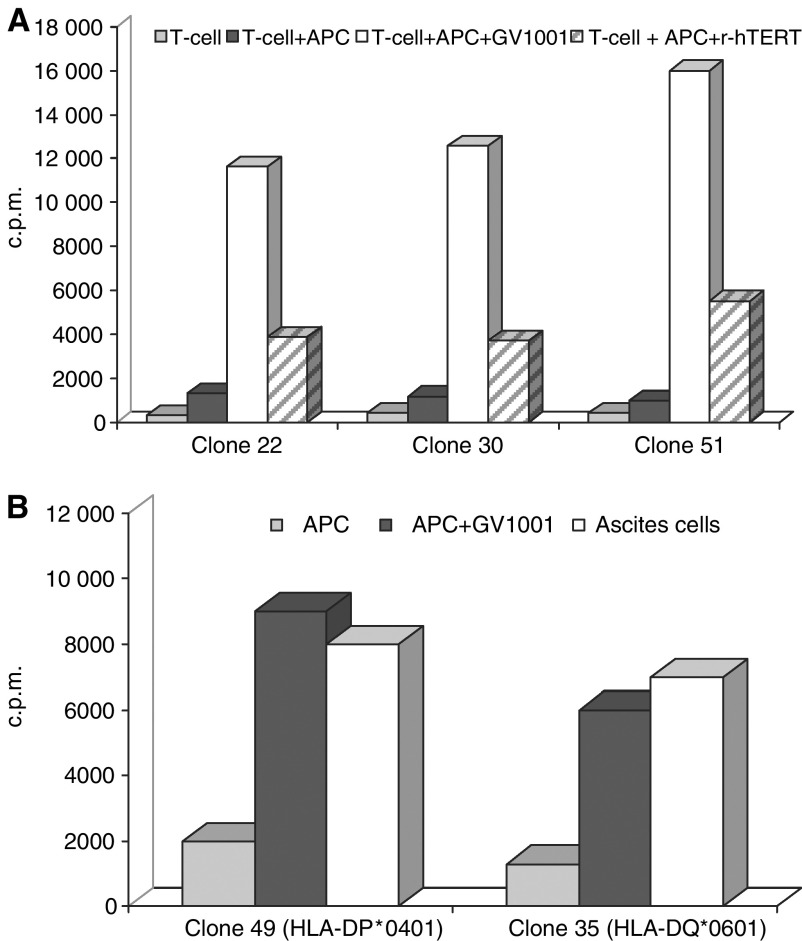
(**A**) Recognition of recombinant hTERT by GV1001-specific T-cell clone nos. 22 and 30 from patient no 102 and clone 51 from patient no 105. T cells (50 000 well^−1^) were stimulated with 50 000 irradiated autologous EBV-transformed B cells as APC for 48 h before labelling and harvesting (see below). The APC were pulsed with either GV1001 (10 *μ*M) or recombinant hTERT (3 *μ*M for clones 22 and 30 and 0.5 *μ*M for clone 51). T cells with APC alone served as negative controls. (**B**) Recognition of ascites cells from patient 105 by T-cell clone nos 35 and 49 derived from the same patient after vaccination. T cells (50 000 well^−1^) were stimulated for 48 h with 50 000 irradiated, T-cell depleted (anti-CD3-coated Dynabeads) ascites cells or 50 000 irradiated autologous EBV-transformed B cells with or without GV1001 (25 *μ*M) as positive and negative controls. After 48 h of culture, ^3^H-thymidine was added and the cultures harvested the next day. Results are expressed as mean c.p.m. of triplicate cultures.

As hTERT may be processed to peptides in many different ways, and the binding motifs of different class II molecules are different, it was important to investigate if different T-cell clones were also capable of recognising hTERT fragments generated by feeding recombinant hTERT to APCs. The three CD4+ Th cell clones derived from patient 102 and 105 all recognised autologous PBMC that were pulsed with recombinant telomerase protein (Figure 6A). As the HLA class II molecules presenting the hTERT epitope to the T-cell clones were different (−DQ, −DR, and −DP) and the three clones have different fine-specificity (results not shown), these results strongly suggest that natural processing of hTERT may give rise to many different peptide fragments, fitting into multiple HLA class II molecules.

For ethical reasons it is difficult to get tumour biopsies from patients with inoperable pancreatic cancer for other than diagnostic purposes. It is also generally difficult to establish even short-term cell lines from such patients, should a biopsy be available. For this reason, we were, with one exception, unable to investigate if GV1001-specific T cells from this series of patients were capable of recognising their autologous tumour cells. Several CD4+ Th clones were expanded from blood sampled from patient no. 105 in week 10 after vaccination. At a later stage patient no. 105 developed ascites and a T-cell depleted mononuclear cell suspension was prepared from the ascites. This cell suspension, also containing tumour cells, was irradiated and tested for its capacity to stimulate autologous GV1001-specific Th cell clones. The results shown in Figure 6B demonstrate that the ascites cells were recognised both by T-cell clones restricted by HLA-DP4 and -DQ6 to the same extent as the autologous APCs pulsed with GV1001. These results strongly indicate that the T cells recognise either a GV1001 equivalent epitope expressed by the tumour cells themselves or cross-presented by APCs present in the ascites fluid.

### Dose-related survival and correlation with immune response

**Figure 7:**
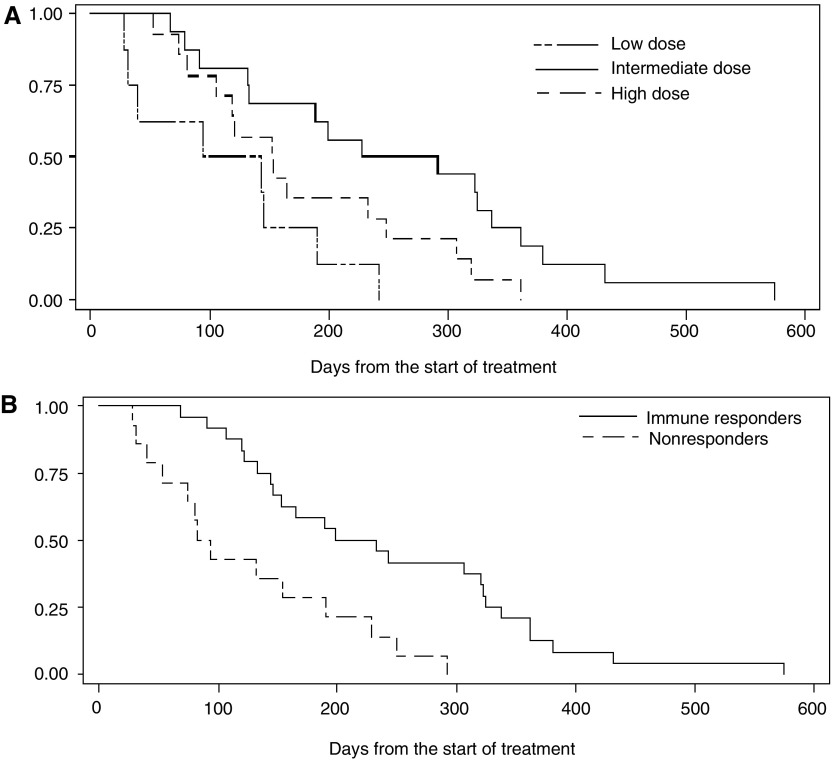
(**A**) Survival (Kaplan–Meier plot) of the evaluable patients treated with different doses of GV1001. Median survival (95% conf. int.): Low-dose group (*n*=8); 119 days (31–190), intermediate dose group (*n*=16); 260 days (133–337), and high-dose group (*n*=14); 153 days (106–249). Intermediate *vs* low dose; *P*=0.006, intermediate *vs* high dose; *P*=0.05 (log-rank). (**B**) Survival (Kaplan–Meier plot) of all immune responders (*n*=24) *vs* all non-responders (*n*=14) in the whole evaluable study population. Median survival (95% confidene interval) was; 216 days (146–323) for the immune responders and 88 days (53–190) for the non-responders. *P*=0.0001 (log-rank).

Although survival was not a primary endpoint in this study, we had the opportunity to follow the included patients for survival, and observed what may be a treatment-related survival benefit. The survival curves for the three dose groups are presented in Figure 7A, demonstrating a significantly increased survival in the intermediate dose group compared to the other groups (intermediate *vs* low: *P*=0.006, intermediate *vs* high: *P*=0.05). The median survival for the low, intermediate, and high-dose groups were, 4.0, 8.6, and 5.1 months, respectively. No statistically significant difference was observed between the low- and high-dose group.

The survival of the whole evaluable study population, divided in immune responders and non-immune responders, is presented as Kaplan–Meier plots in Figure 7B which clearly demonstrates that the immune responders live longer than the non-responders. The observed median survival was 7.2 months for the responders and 2.9 months for the non-responders (*P*=0.001).

## DISCUSSION

We here present results of the first clinical test of a novel, broadly applicable, peptide-based cancer vaccine. The data show that vaccination with GV1001 is safe and induces dose related cellular immune responses that correlate with prolonged survival for treatment naïve non-resectable pancreatic cancer patients. Telomerase vaccination has also been demonstrated to be safe by other groups reporting clinical results from testing of hTERT mRNA-transfected dendritic cell vaccination of patients with prostate cancer (Su *et al*, 2005), and from vaccination of patients with advanced cancer with an HLA class I A2 allele-specific telomerase peptide (Parkhurst *et al*, 2004; Vonderheide *et al*, 2004).

The finding that 75% of the patients in the intermediate dose group developed a T-cell response to the vaccine supports the notion that GV1001 contains binding motifs that allows promiscuous binding to a broad array of HLA class II molecules. This could be shown by cloning T cells from two of the responders and testing their HLA restriction (Figure 1). This property makes it unnecessary to tissue-type patients for HLA compliance as an inclusion criterion, which is characteristic for many other HLA allele-specific peptide cancer vaccines (i.e. HLA class I epitopes).

Our results show that survival correlates with vaccine dose in a statistically significant manner. The median survival for the intermediate dose group, the group with the largest fraction of immune responders, was 8.6 months, which was significantly higher than for the two other dose groups. However, this is a small non-randomised study and statistically significant differences between groups must be interpreted with caution.

For the overall evaluable study population the median survival for the immune responders (7.2 months) was significantly higher than for the non-responders. The non-responder group will contain patients with a shorter life expectancy than in the immune responder group and many of these patients die within the first 90 days. However, it is interesting to observe that after 300 days, only patients in the immune responding group are still alive. Booster vaccination after end of study may have contributed to the survival benefit by maintaining an anticancer immune response in some of patients. Considering that non-resectable pancreatic cancer patients normally are strongly immunosuppressed and have a short life expectancy, the results are very encouraging. Gemcitabine treatment, which is the standard treatment in many countries, generally results in a median survival of 4.8–5.6 months (Burris *et al*, 1997; Storniolo *et al*, 1999).

One intriguing finding was that vaccination with the high dose resulted in detection of a lower number of DTH responders than in the intermediate dose group. An explanation may be that a very high local concentration of peptide (5 mM) may result in leakage of peptide to surrounding tissue, uptake, and presentation of vaccine by non-professional APCs. This can result in anergy of the specific T cells or selection of T cells with low-affinity TcR or polarisation of the T-cell response towards a Th2 response (Boyton and Altmann, 2002) The peptide dose–response curve of the T-cell clone depicted in Figure 5 demonstrate that high doses of peptide results in a reduced T-cell response *in vitro*, indicating that similar effects may be responsible for the weaker response to the high dose after vaccination. This biphasic curve also has implications for the interpretation of the immune responses observed. It is not only conceivable that some samples that were scored negative would have been positive with a higher peptide dose than that used in the assay (25 *μ*M), but also the opposite scenario is conceivable. Some samples could have scored negative because the peptide dose used was too high.

GV1001 was capable of inducing CD4+ T helper (Th) cells in the majority of the evaluable patients. These CD4+ T cells were capable of recognising APCs pulsed with recombinant hTERT and in one patient we also demonstrated that they recognised ascites derived cells, clearly demonstrating that both exogenously and endogenously derived hTERT is processed and presented in the context of HLA class II molecules. The mechanism of action of Th cells in the antitumour response is complex and different from that of tumour-specific CTLs. As most tumour cells do not express HLA class II molecules, direct recognition of tumour cells does not take place unless expression of class II molecules have been induced by inflammatory cytokines locally. The cytokine profile generated by the patients T cells after vaccination with GV1001 is highly compatible with such a scenario, as both IFN*γ* and TNF*α* was produced (Figure 4A and B). Thus a supply of relevant cytokines for polarisation of the T-cell response in the direction of a general antitumour inflammatory response, giving rise to indirect killing by CD4+ cells in HLA class II-negative tumours (Hung *et al*, 1998; Janssen *et al*, 2003) may be part of the mechanism of action. The observation of production of nanogram levels of IL13 is interesting. Production of similar levels of IL13 in T cells *in vitro* has been shown to be prognostic for the development of acute graft-versus-host disease in patients who have received unrelated donor stem cell transplantation (Jordan *et al*, 2004). Taken together these data indicate that vaccination may direct potent inflammatory response to the tumour itself or to tumour draining lymph nodes. Induction of tumour-specific Th cells is considered also to be mandatory for establishing and maintaining a clinically efficient anticancer immune response caused by CTLs (Mumberg *et al*, 1999; Schurmans *et al*, 2001) Vaccine induced Th cells, may interact with professional APCs (i.e. dendritic cells) that have engulfed dead tumour tissue, *in situ* in the tumour or in draining lymph nodes. This interaction may give rise to a ‘second wave’ of tumour reactive T cells, including CD8+ CTLs, that are directed against unknown tumour/tissue-specific antigens (cross presentation) (Markiewicz *et al*, 2001). Furthermore Th cells also play an important role in overcoming the immune-suppression caused by regulatory CD4+CD25+ T cells (Casares *et al*, 2003).

The results encourage further and larger controlled clinical studies with the intermediate dose of telomerase vaccine, GV1001, in order to document the clinical benefits of the treatment indicated by this study. The results should also open up for clinical testing in patients with less tumour burden and longer life expectancy than patients with advanced pancreatic cancer.

## Figures and Tables

**Table 1:** Characteristics, summary of treatment and immune responses, and survival for the individual patients

		**Characteristics (baseline)**			**Treatment weeks 1–12**		**Follow-up and survival**		
**Dose group**	**Patient No.**	**Age (sex)**	**KPS (%)**	**Tumour site/metastasis^a^ (+/−)**	**No. of Injections/ Completed study**	**Immune-responses DTH/T cells**	**No. of booster injections**	**DTH**	**Survival days from start of treatment**
Low	1	69 (M)	85	cap/+	6/no	−/−	—	—	28
	2	69 (M)	100	cap/−	8/yes	+/−	—	—	144
	3	64 (M)	80	cap, cor/−	7/no	−/−	—	—	40
	4	61 (F)	70	cap/+	8/yes	−/−	—	—	190
	5	61 (M)	100	cau, cor/+	6/no	−/−	—	—	31
	7^b^	60 (F)	85	cau, cor/+	5/no	−/−	—	—	20
	8	59 (F)	85	cap/+	8/yes	+/−	—	—	146
	9^c^	37 (M)	85	cor/+	6/no	−/−	—	—	94
	10^b^^,^^d^	59 (F)	100	cap/−	4/no	−/−	—	—	290
	11^b^	47 (M)	70	?/+	1/no	−/−	—	—	3
	12	59 (M)	100	cap/−	8/yes	+/−	3	−	243
									
Intermediate	101	73 (F)	95	cap/+	8/yes	+/−e	6	+	325
	102	55 (F)	95	cor, cau/−	8/yes	+/+	7	+	362
	103	58 (F)	95	cap/+	8/yes	+/−	2	−	133
	104	64 (F)	95	cap/−	8/no	−/+	—	—	68
	105	58 (M)	95	cap/+	8/yes	+/+	3	+	189
	106	49 (M)	85	cor/+	7/no	−/−	—	—	80
	107	73 (M)	85	cau/+	8/no	−/−	—	—	132
	108	56 (M)	95	cap/+	8/yes	+/+	10	+	381
	109^c^	68 (M)	95	?/?	6/no	−/nt	—	—	228
	110	59 (M)	90	cor, cau/−	8/yes	−/−	4	−	292
	111	68 (M)	100	cap/−	8/yes	−/+	2	−	337
	112	67 (F)	95	cap/−	8/yes	+/+	1	−	199
	113	58 (M)	95	cap/−	8/yes	−/+	2	+	432
	114^b^	71 (F)	95	cau/+	5/no	−/nt			23
	115	60 (F)	90	cap/−	8/yes	+/+	13	+	575
	116	66 (F)	95	cau/−	8/yes	+/+	6	−	323
	117	69 (M)	90	cap/−	8/yes	+/−	—	—	91
									
High	201	45 (F)	80	cap/−	10/yes	+/+	—	—	320
	202	58 (M)	85	cor, cau/−	8/yes	+/+	—	—	165
	203^b^	54 (F)	85	cap/+	2/no	−/nt	—	—	27
	204	70 (F)	90	cap/+	7/no	−/nt	—	—	53
	205^b^	42 (F)	70	cap/+	4/no	−/nt	—	—	10
	206^b^	49 (M)	75	cor, cau/+	4/no	−/nt	—	—	12
	207	58 (F)	95	cap/−	8/no	−/−	—	—	82
	208	75 (M)	95	cap/−	8/yes	−/+	6	−	307
	209	54 (M)	80	?/?	8/yes	−/+			121
	210	53 (F)	85	cor/+	7/no	−/−			75
	211	73 (M)	95	cor/−	8/yes	−/−	4	−	249
	212^b^^,^^f^	40 (F)	90	cor/−	8/yes	−/+	1	−	120
	213^b^^,^^f^	68 (F)	95	cap/−	8/yes	+/+	3	−	176
	214	56 (M)	85	cor/−	8/yes	−/−	2	+	154
	215	64 (M)	90	cap/−	8/yes	−/+	—	—	119
	216	58 (M)	90	cap/−	8/yes	−/+	1	−	153
	217^b^	63 (M)	75	cap/+	4/no	−/−	—	—	12
	218	46 (M)	90	cap/−	8/yes	−/+	—	—	106
	219	72 (F)	95	cor/+	8/yes	−/+	8	−	365
	221	70 (F)	95	cap, cor/−	8/yes	+/+^e^	5	+	233
									

DTH=delayed-type hypersensitivity; KPS=Karnofsky performance status; nt=not tested; na=not applicable; cap=caput; cau=cauda; cor=corpus; ?=unknown.

a 81% of patients with metastasis had liver metastasis.

b Not evaluable.

c Withdrawn to other treatment after week 4.

d Withdrawn to surgery.

e Positive at base line.

f Earlier resected patient.
